# Inhibitory Effect of r-Hirudin Variant III on Streptozotocin-Induced Diabetic Cataracts in Rats

**DOI:** 10.1155/2013/630651

**Published:** 2013-12-10

**Authors:** Xiaojian Gong, Qiuyan Zhang, Shuhua Tan

**Affiliations:** ^1^Department of Pharmacology, School of Pharmacy, China Pharmaceutical University, Nanjing 210009, China; ^2^State Key Laboratory of Natural Medicines, School of Life Science and Technology, China Pharmaceutical University, Nanjing 210009, China

## Abstract

The *in vivo* inhibitory effect of r-hirudin variant III (rHV3) on streptozotocin (STZ)-induced diabetic cataracts in rats was investigated. SD-rats were firstly made diabetic by a single intraperitoneal injection of 2% (W/V) STZ (65 mg/kg). Two weeks later, cataract formation was examined by slit lamp microscope, and the cataracted animals were randomly grouped. The animals in the treated groups received rHV3 drops administration to the eyes with various doses. After 4 weeks treatment, the animals were sacrificed to evaluate the biochemical changes of aldose reductase (AR), superoxide dismutase (SOD), glutathione peroxidase (GSH-Px), and malondialdehyde (MDA) levels in the eye lens. Meanwhile, the cataract progression was monitored by slit lamp microscope. As a result, rHV3 drops treatment significantly increased the activities of SOD and GSH-Px in the lens in a dose-dependent manner, whereas AR activity and MDA level in the lens were dramatically decreased. Also, the morphological observation further confirmed the inhibition of the development of STZ-induced diabetic cataracts by the rHV3 drops treatment. Thus, our data suggest that rHV3 drops are pharmacologically effective for the protection against STZ-induced diabetic cataracts in rats.

## 1. Introduction

Cataract, characterized by cloudiness or opacification of the eye lens, is the leading cause of visual disability and blindness worldwide, and it accounts for approximately 50% of all blindness [1, 2]. Although cataract is a multifactorial optic disorder associated with various risk factors, such as malnutrition, drugs, and UV light exposure, however, aging and diabetes are the most significant contributors [3, 4]. Also, clinical, epidemiological and basic research studies have shown that diabetes is associated with the cataract formation [5]. Due to the widespread prevalence of diabetes worldwide, the incidence of diabetic cataracts has been steadily increased. The only treatment of diabetic cataract is surgery so far; however, it may lead to serious postoperative complications such as corneal edema and increased intraocular pressure. Therefore, it has been highly desirable to develop effective therapeutic agents for the prevention and the treatment of diabetic cataracts.

Cataractogenesis is one of the earliest secondary complications of diabetes mellitus and multiple mechanisms are involved in this process, which mainly include activation of the aldose reductase (AR)/polyol pathway, oxidative stress, and nonenzymatic glycation of lens fibers [1]. Also, the polyol pathway plays a central role as the initiating factor in diabetic cataract formation [5], and it also activates the diabetes induced oxidative stress in the lens [6].

To date, a number of AR inhibitors (ARI) have been developed for the treatment of diabetic cataract. For example, a proprietary formulation of an ARI improved functional vision in diabetic dogs with early lens opacities [7]. The fidarestat, a potent and specific ARI, was effective in the prevention and treatment of diabetes-associated cataract formation in streptozotocin (STZ)-diabetic rats [8]. The KIOM-79, a combination of four plant extracts, inhibited the development of diabetic cataract through the inhibition of AR in the lenses of Zucker diabetic fatty (ZDF) rats, an animal model of type 2 diabetes [9]. The isoquinoline alkaloids derived from *Tinospora cordifolia* stem showed potential anticataract activity by inhibiting AR and polyol accumulation in rat lens *in vitro *[10]. The neochlorogenic acid (5-O-caffeoylquinic acid, CA), a phenolic compound found ubiquitously in plants showed the inhibitory effect on diabetic cataractogenesis through the inhibition of AR activity [11].

Apart from ARIs, multiple antioxidants appear to be potentially useful in the treatment of diabetic cataract because diabetes increases the oxidative stress, which is thought to play an important role in diabetic cataractogenesis [6]. Alpha-lipoic acid (ALA) is a powerful antioxidant, and it has been proved that dihydrolipoate-LA treatment delays the development and progression of cataract in rats with STZ-induced diabetes [12]. Besides, antioxidant protein 2 (AOP2) has been shown to protect human lens epithelial cell (hLEC) from oxidative stress and possesses the potential to prevent hyperglycemia-mediated diabetic cataracts in diabetes [13].

Hirudin is a typically secreted small polypeptide derived from the salivary gland of the medicinal leech* Hirudo medicinalis*. It includes three principal hirudin variants comprised of 65~66 amino acids, which are designated HV1, HV2, and HV3 [14]. So far, two hirudin variants and a hirudin analogue have been approved by the FDA for marketing as an antithrombotic drug [15, 16]. In addition, it has been proved that rHV3 might protect against galactose-mediated lens epithelial cells damage *in vitro* by up-regulating antioxidant enzymes, down-regulating AR activity, and inhibiting lens epithelial cell apoptosis through the mitochondrial pathways [17–19]. Since the STZ-induced diabetic rat is a well established model of the chronic complications of human diabetes [20], in this study the experiments were designed to further evaluate the *in vivo* protective effect of rHV3 eye drops on STZ-induced diabetic cataracts in rats.

## 2. Materials and Methods

### 2.1. Materials

STZ and NADPH (nicotinamide adenine dinucleotide phosphate) were purchased from Sigma Chemical Company (St. Louis, MO, USA). BCA protein assay kit was obtained from Biouniquer Technology Co., LTD (USA). Kits for superoxide dismutase (SOD), glutathione peroxidase (GSH-Px), and MDA (Malondialdehyde) quantification were obtained from Nanjing Jiancheng Bioengineering Institute (Nanjing, China). Sodium Hyaluronate (NaHA) was obtained from Shandong Freda Biopharm Co., LTD (Jinan, China), Pirenoxine sodium eye drops (0.8 mg pirenoxine sodium in 15 mL vehicle solution) were purchased from Wuhan Grand Pharmaceutical Group Co., Ltd (Wuhan, China). All other chemicals and solvents were of analytical grade and were obtained from local companies.

### 2.2. Expression and Purification of rHV3

The purified rHV3 was prepared as described previously [21]. Briefly, the recombinant *E. coli* harboring pTASH [22] was cultivated in a 7-l bioreactor under the optimal fed-batch fermentation condition, and the expressed product was excreted into the culture supernatant. After collection of the supernatant, a four-step procedure was applied to the product purification, which included ultrafiltration, hydrophobic chromatography, anion-exchange chromatography, and preparative reversed-phase fast protein liquid chromatography (FPLC). The finally purified product was lyophilized and stored at −20°C.

### 2.3. Preparation of rHV3 Eye Drops

Firstly, the vehicle of eye drops was prepared. For 100 mL of vehicle solution, 0.1 g NaHA (~1.3 × 10^6^ Kd) was completely dissolved in 75 mL of dd H_2_O by stirring at 60~80°C. Subsequently, 1.116 g boric acid, 0.191 g sodium tetraborate, 0.22 g sodium chloride, 0.5 g EDTA-Na_2_·2H_2_O, and 0.03 g ethyl-p-hydroxybenzoate were sequentially added and stirred, subsequently supplemented with dd H_2_O to 100 mL. In this formula, NaHA was used as viscous vehicle in order to improve the residence of rHV3 eye drops in the tear film [23]. For rHV3 eye drops preparation, the finally purified rHV3 product was dissolved in above vehicle solution at various concentrations of 500, 1000, and 2000 ATU/mL, respectively, and adjusted to pH 7.4, as well as filtered through a 0.22 *μ*m filter membrane. Here, one ATU corresponds to approximately 0.1 *μ*g rHV3 as the specific activity of finally purified rHV3 is approximately 10000 ATU/mg.

### 2.4. Animal Modeling and Drug Administration

Totally 184 male S-D rats at age of ~5 weeks after birth with an average body weight of 150 ± 20 g were purchased from Shanghai Super-B&K laboratory animal Corp. Ltd (Shanghai, China), and housed in an air-conditioned animal house under a normal day/night cycle and fed a normal rodent chow diet.

A group of rats which received only the vehicle served as a control group (group A, *n* = 16). Other animals in experimental groups were overnight-fasted, then diabetes was induced by a single intraperitoneal injection of 2% (W/V) STZ (65 mg/kg) in 0.02 M citrate buffer (pH 4.5). Seventy-two hours after STZ injection, fasting blood glucose levels were measured and animals with blood glucose levels >11 mmol/L were considered diabetic. Two weeks after the induction of diabetes, the cataract formation was examined by slit lamp microscope (YZ5E model, 66 Vision-Tech Co., Ltd., Suzhou, China). The noncataractous animals were eliminated, and the cataractous animals were randomly divided into the following 5 groups (*n* = 16): B (cataractous rats treated with 50 *μ*L of eye drops vehicle solution), C (cataractous rats treated with 50 *μ*L of 0.5 ATU/*μ*L rHV3 eye drops), D (cataractous rats treated with 50 *μ*L of 1 ATU/*μ*L rHV3 eye drops), E (cataractous rats treated with 50 *μ*L of 2 ATU/*μ*L rHV3 eye drops), and F (cataractous rats treated with 50 *μ*L of 0.053 *μ*g/*μ*L pirenoxine sodium eye drops as the manufacturer suggested). Drug administration was performed by instillation of 50 *μ*L eye drops or vehicle solution to each eye, 3 times per day and consecutively for four weeks. The body weight of the rats was recorded every week. All the procedures were carried out in compliance with the Regulations of Experimental Animal Administrations issued by the State Committee of Science and Technology of the People's Republic of China.

### 2.5. Slit Lamp Microscope Examination and Cataract Classification

Eyes were examined every week using a YZ5E model slit lamp biomicroscope (66 Vision-Tech Co., Ltd., Suzhou, China). Initiation and progression of lenticular opacity was graded according to the classification of lens opacification [24–26]: grade 0, clear lens or no vacuoles present; grade 1, peripheral vesicles; grade 2, vacuoles located at the periphery of the lens occupying an area of between one-third and two-thirds of the radius from the periphery; grade 3, vacuoles extending up to two-thirds of the radius from the periphery and nuclear opacity may be seen; grade 4, mature cataract with nuclear and peripheral cortex opacities; grade 5, hypermature cataract with whole lens opacity.

### 2.6. Lens Collection and Processing

After four weeks treatment, animals were sacrificed by decapitation and their eyeballs were removed for biochemical analysis. The lenses were dissected by the posterior approach [27]. Briefly, a small incision was made on the posterior side of the eye using the scissors. The lenses were collected by pressing with tweezers against the side of the eye opposite of the incision and stored at −70°C for subsequent analysis. A 10% homogenate was prepared from 2 pooled lenses of each rat in ice-cold neutral normal saline in Eppendorf tube, then centrifuged at 10 000 g for 15 min at 4°C. The clear supernatant was used for subsequent biochemical analysis.

### 2.7. Biochemical Analyses

The activities of SOD and GSH-Px were determined by using the commercial kits. Serum glucose and lens MDA were measured by the glucose oxidase-peroxidase (GOD-POD) and the thiobabituric acid (TBA) methods, respectively, according to the guidelines of the commercial kits. AR activity was measured using the protocol described by Kador and colleagues [28, 29] with slight modification. Briefly, the assay was conducted on a UV-1600 spectrophotometer by following the decrease in the absorbance of NADPH at 340 nm over a 5-min period with dL-glyceraldehydes as the substrate. The assay mixture in 1 mL of 100 mmol/L potassium phosphate buffer (pH 6.2) contained 0.4 M (NH_4_)_2_SO_4_, 80 *μ*mol/L NADPH, enzyme extract, and 5 mmol/L dL-glyceraldehyde. Meanwhile, an appropriate control which contained all of the above-mentioned components except dL-glyceraldehyde was setup to correct for nonspecific NADPH reductase activity. The reaction was conducted at 30°C, initiated by the addition of NADPH, and terminated by the addition of 1 mL of cold 0.5 mol/L HCl. The enzyme activity was expressed as the amount (*μ*mol) of NADPH oxidized per minute by 1 mg of protein.

### 2.8. Histological Analysis

Lenses from diabetic cataract rats were fixed in 10% formalin overnight, embedded in paraffin, sectioned (4-5 *μ*m in thickness), and stained with hematoxylin and eosin.

### 2.9. Statistical Analysis

Data were represented as mean ± standard deviation (SD). Differences between groups were assessed by ANOVA using the SPSS software package for Windows. Post hoc testing was performed for intergroup comparisons using the Bonferroni test. Statistical analysis of the average of the cataract score of the lens opacity was performed using the Kruskal-Wallis.  *P* < 0.05 was considered as significantly altered.

## 3. Results

### 3.1. Diabetic Cataract Incidence

**Figure 1:**
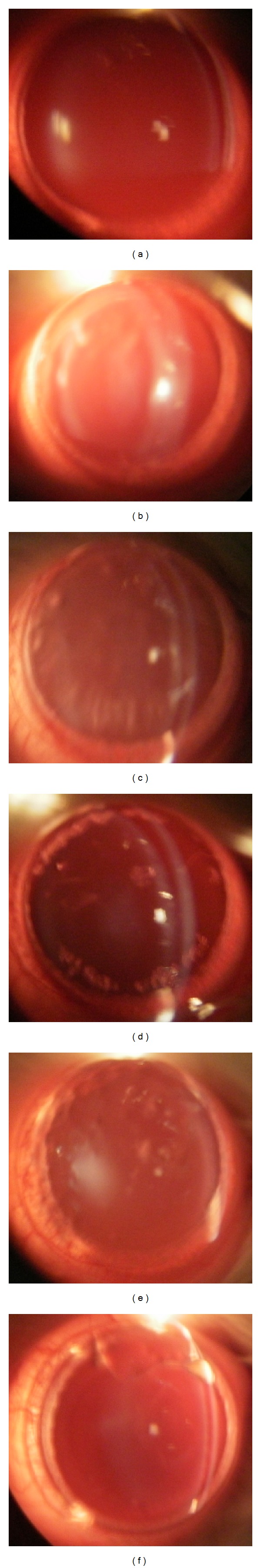
Two weeks after diabetic modeling by STZ injection, the onset of diabetic cataracts in rats before rHV3 eye drops administration. (a): normal control (group A); (b): diabetic cataracts to be untreated (group B); (c): diabetic cataracts to be treated with 50 *μ*L (one drop) of 0.053 *μ*g/*μ*L pirenoxine sodium eye drops each time (group F); (d), (e), and (f): diabetic cataracts to be treated with 50 *μ*L (one drop) of 0.5, 1, 2 ATU/*μ*L rHV3 eye drops each time, respectively (groups C, D, E).

SD-rats were made diabetic after 72 hours of intraperitoneal injection of STZ (65 mg/kg) with fasting blood glucose levels >11 mmol/L. Two weeks after diabetic modeling by STZ injection, cataract was developed due to hyperglycemia in approximately 92% of lenses in diabetic rats. The body weight of diabetic cataractous rats was dramatically decreased, and some peripheral vesicles were observed in the lenses (Table 1 and Figure 1), which indicated the formation of diabetic cataracts.

### 3.2. Effect of rHV3 Drops on the Cataractous Lens of STZ Induced Diabetic Rats

**Figure 2:**
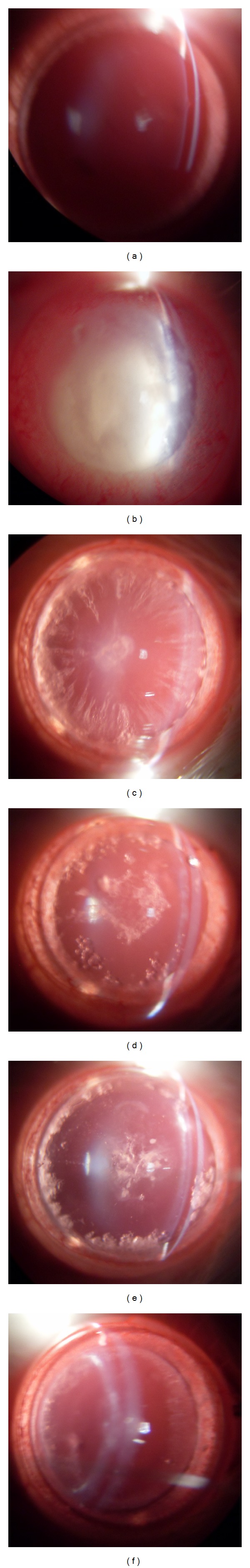
Inhibitory effect of rHV3 eye drops on STZ-induced diabetic cataracts in Sprague-Dawley rats. Representative photographs of lens from each group at the end of four weeks rHV3 eye drops treatment. (a): normal control (group A); (b): diabetic cataracts untreated (group B); (c): diabetic cataracts treated with 50 *μ*L (one drop) of 0.053 *μ*g/*μ*L pirenoxine sodium eye drops each time (group F); (d), (e), and (f): diabetic cataracts treated with 50 *μ*L (one drop) of 0.5, 1, 2 ATU/*μ*L rHV3 eye drops each time, respectively (groups C, D, E). The drug was administrated 3 times per day for four weeks.

After four weeks treatment with rHV3 eye drops, the cataract scores in each experimental group are summarized in Table 2, and the representative images of the lenses from each group are shown in Figure 2. The lenses of normal control rats (group A) appeared to be clear and free of opacities throughout the experimental period. The progression of cataract appeared to be inhibited in all the rHV3 eye drops treated groups (groups C, D, E) as compared to the untreated cataractous rats (group B), and there was a statistically significant difference between group B and the rHV3 eye drops treated groups (groups C, D, E) (*P* < 0.01), and between groups B and F (*P* < 0.05). Also, there was a statistically significant difference between the rHV3 eye drops treated groups (groups C, D, E) and the pirenoxine sodium eye drops treated group (group F) (*P* < 0.01). These data indicated that administration of rHV3 eye drops might dose-dependently inhibit the development of STZ induced diabetic cataract, and the effect was significantly more pronounced than that with pirenoxine sodium eye drops treatment.

### 3.3. Effect of rHV3 Drops on the Polyol Pathway

The data in Table 3 showed that the level of AR, the key enzyme of the polyol pathway, was dramatically inhibited in the rHV3 eye drops treated groups (groups C, D, E). There were statistically significant differences between groups B and E (*P* < 0.01). Moreover, high-dosage treatment with rHV3 eye drops seemed to show better inhibitory effect on AR activity than that with the pirenoxine sodium eye drops (Table 3).

### 3.4. Effect of rHV3 Drops on the Oxidative Stress and the Antioxidant System

The lens MDA levels in untreated cataractous group (group B) were significantly increased as compared to the normal control (group A), which implied the increased lipid peroxidation in the lens due to STZ-induced diabetic cataract. However, the MDA levels in groups C, D, E were significantly decreased with rHV3 drops treatment as compared to group B, and there were statistically significant differences between groups B and E (*P* < 0.01) and groups B and D (*P* < 0.05) (Table 4).

The specific activity of SOD was remarkably increased with rHV3 drops treatment as compared to group B, and there were statistically significant differences between all the treated cataractous groups (C, D, E, F) and the untreated cataractous group (B) (*P* < 0.01) (Table 4). In addition, the specific activities of GSH-Px in groups D and E were significantly increased as compared to group B (*P* < 0.01). Moreover, high-dosage treatment with rHV3 eye drops showed better enhancing effect on GSH-Px activity than that with the pirenoxine sodium eye drops (*P* < 0.01) (Table 4).

### 3.5. Histological Analysis

**Figure 3:**
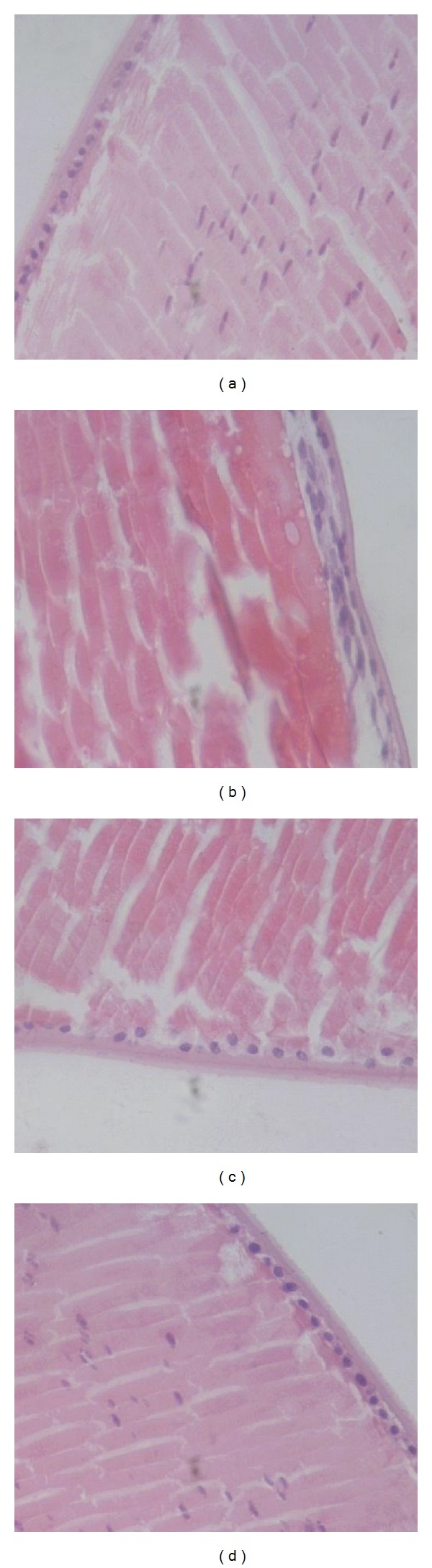
Histological examination of lenses of cataractous rats after 72 hours treatment with rHV3 eye drops. (a): normal control (group A); (b): diabetic cataracts untreated (group B); (c) and (d): diabetic cataracts treated with 50 *μ*L (one drop) of 0.5, 2 ATU/*μ*L rHV3 eye drops, respectively.

The result of histological analysis was shown in Figure 3. In normal control group (group A) the cellular architecture of the lenses was orderly arranged. However, in the untreated diabetic cataractous rats (group B), cortical fiber cell swelling and vacuoles in the cortical fiber region were observed, also the number of the nucleus remarkably decreased. In group C, which was treated with 50 *μ*L (one drop) of 0.5 ATU/*μ*L rHV3 eye drops, cortical fiber cell swelling was attenuated, no vacuoles were observed, also the decrease in the number of nucleus was alleviated as compared to group B. In group E, which was treated with 50 *μ*L (one drop) of 2 ATU/*μ*L rHV3 eye drops, histological pathological changes of lens fiber were effectively prevented.

## 4. Discussion

In this study, the data demonstrated for the first time that rHV3 eye drops treatment might *in vivo* effectively inhibit the development of STZ-induced diabetic cataract in rats.

As for the mechanisms of cataract development in diabetes, it has been revealed that activated polyol pathway in glucose disposition, oxidative stress, and increased formation of advanced glycation end products are involved [1, 30]. Also, the activated polyol pathway is likely a major contributor to hyperglycemia-induced oxidative stress, and they may synergistically contribute to the development of lens opacity in hyperglycemia [3, 6, 30]. Thus, inhibition of AR may prevent or delay the development of the diabetic cataract [5, 9, 31, 32]. It has been proved *in vitro* and *in vivo* that both ARIs and antioxidants were beneficial in the prevention or treatment of this diabetic complication [5].

Based on previous *in vitro* studies, which indicated that rHV3 might protect against galactose-mediated lens epithelial cells damage in human lens epithelial cell line [17] and primary rat lens epithelial cells (rLECs) [19], we evaluated the *in vivo* inhibitory effect of rHV3 drops on STZ-induced diabetic cataracts in Sprague-Dawley rats by biochemical and morphological approaches.

It was revealed that rHV3 eye drops treatment could dose-dependently inhibited the AR in the lens, and even high-dosage rHV3 treatment showed better inhibitory effect on AR activity than pirenoxine sodium eye drops. In addition, as the MDA level in the lens is usually a representative biomarker that is assessed to examine the level of lipid peroxidation, and SOD and GSH-Px are major antioxidant enzymes that may protect from oxidative damage due to their respective abilities of catalyzing superoxide anions into hydrogen peroxide and reducing organic peroxides and hydrogen peroxide to nontoxic products, we detected the effect of rHV3 drops on the MDA level, SOD and GSH-Px activities in the lens. The data indicated that the MDA accumulation in the lens could be decreased in a dose-dependent manner with r-HV3 drops treatment, while the SOD and GSH-Px levels in the lens were enhanced dose-dependently with r-HV3 drops treatment. These results implied that rHV3 eye drops treatment might protect against oxidative stress that caused damage to the lens.

In addition to biochemical analyses, histological examination, and morphological observation further verified that rHV3 eye drops might effectively inhibit the development of STZ induced diabetic cataract, and the effect was significantly more pronounced than that with pirenoxine sodium eye drops treatment.

Taken together, our data suggest that rHV3 drops are pharmacologically effective *in vivo* for the protection against STZ-induced diabetic cataracts in rats, and the possible mechanism is likely due to its ability to inhibit the activation of polyol pathway and protect against oxidative stress by enhancing the antioxidant enzyme activities.

## Figures and Tables

**Table 1:** The body weight and lens opacity of STZ-induced diabetic cataract in rats before rHV3 eye drops treatment ($\bar{x}\pm s$).

Group	*n*	Body weight (g)	Lens opacity
A	16	249.63 ± 14.15**	0 ± 0**
B	16	177.56 ± 17.60	1.44 ± 0.51
C	16	173.00 ± 11.07	1.50 ± 0.52
D	16	169.63 ± 13.98	1.38 ± 0.5
E	16	164.75 ± 18.14	1.81 ± 0.40
F	16	166.50 ± 16.82	1.69 ± 0.48

**P < 0.01 versus group B (model, treated with eye drops vehicle).

**Table 2:** The body weight and lens opacity of cataractous rats after four weeks treatment with rHV3 eye drops ($\bar{x}\pm s$).

Group	*n*	Body weight (g)	Lens opacity
A	16	360.69 ± 31.63**	0.19 ± 0.40^∗∗##^
B	13	170.85 ± 28.65	3.69 ± 0.63
C	13	170.15 ± 26.70	2.00 ± 0.82^∗∗##^
D	14	164.93 ± 14.58	1.86 ± 0.53^∗∗##^
E	15	166.73 ± 32.30	1.73 ± 0.46^∗∗##^
F	12	171.25 ± 29.89	3.00 ± 0.85

**P < 0.01 versus group B (model, treated with eye drops vehicle).

^##^ *P* < 0.01 versus group F (pirenoxine treated).

**Table 3:** The effect of rHV3 eye drops on the activity of AR in the lens on diabetic cataract in Sprague-Dawley rats ($\bar{x}\pm s$).

Group	*n*	AR (mU/mg protein)
A	8	8.30 ± 0.48^∗∗##^
B	8	19.38 ± 1.37
C	8	17.88 ± 2.8
D	8	17.05 ± 1.98
E	8	14.17 ± 1.23**
F	8	16.53 ± 1.87*

*P < 0.05, **P < 0.01 versus group B (model, treated with eye drops vehicle).

^#^ *P* < 0.05, ^##^ *P* < 0.01 versus group F (pirenoxine treated).

**Table 4:** The effect of rHV3 eye drops on the activities of MDA, SOD, and GSH-Px in the lens on diabetic cataract in Sprague-Dawley rats ($\bar{x}\pm s$).

Group	*n*	MDA (nMol/mg prot)	SOD (U/mg prot)	GSH-Px (U/mg prot)
A	16	0.441 ± 0.07**	10.07 ± 1.77^∗∗##^	8.55 ± 1.83**
B	13	0.686 ± 0.16	4.75 ± 1.38	4.03 ± 1.31
C	13	0.513 ± 0.20	6.92 ± 1.52**	5.18 ± 1.77
D	14	0.482 ± 0.21*	7.84 ± 1.29**	7.84 ± 1.81**
E	15	0.456 ± 0.14**	8.21 ± 1.31**	9.73 ± 2.50^∗∗##^
F	12	0.478 ± 0.15*	7.08 ± 1.17**	6.82 ± 1.41**

*P < 0.05, **P < 0.01 versus group B (model, treated with eye drops vehicle).

^#^ *P* < 0.05, ^##^ *P* < 0.01 versus group F (pirenoxine treated).
